# Oxaliplatin induces the PARP1-mediated parthanatos in oral squamous cell carcinoma by increasing production of ROS

**DOI:** 10.18632/aging.202386

**Published:** 2021-01-20

**Authors:** Dongfang Li, Yuying Kou, Yuan Gao, Shanshan Liu, Panpan Yang, Tomoka Hasegawa, Rongjian Su, Jie Guo, Minqi Li

**Affiliations:** 1 Department of Bone Metabolism, School and Hospital of Stomatology, Cheeloo College of Medicine, Shandong University and Shandong Key Laboratory of Oral Tissue Regeneration and Shandong Engineering Laboratory for Dental Materials and Oral Tissue Regeneration, Jinan 250012, China; 2 Department of Developmental Biology of Hard Tissue, Graduate School of Dental Medicine, Hokkaido University, Sapporo 060-8586, Japan; 3 Life Science Institute of Jinzhou Medical University, College of Basic Medicine of Jinzhou Medical University, Cell Biology and Genetic Department of Jinzhou Medical University, Key Lab of Molecular and Cellular Biology of the Education Department of Liaoning Province, Jinzhou 121001, China

**Keywords:** oxaliplatin, parthanatos, oral squamous cell carcinoma, ROS

## Abstract

Oral squamous cell carcinoma (OSCC) is one of the most common malignant tumors worldwide, and its prognosis is still not optimistic. Oxaliplatin is a type of platinum chemotherapeutic agent, but its treatment effects on OSCC and molecular mechanisms have not been fully elucidated. Parthanatos, a unique form of cell death, plays an important role in a variety of physiological and pathological processes. This study aims to investigate whether oxaliplatin inhibits OSCC by inducing parthanatos. Our results showed that oxaliplatin inhibited the proliferation and migration of OSCC cells *in vitro*, and also inhibited the tumorigenesis *in vivo*. Further experiments proved that oxaliplatin induced parthanatos in OSCC cells, characterized by depolarization of the mitochondrial membrane potential, up-regulation of PARP1, AIF and MIF in the nucleus, as well as the nuclear translocation of AIF. Meanwhile, PARP1 inhibitor rucaparib and siRNA against PARP1 attenuated oxaliplatin-induced parthanatos in OSCC cells. In addition, we found that oxaliplatin caused oxidative stress in OSCC cells, and antioxidant NAC not only relieved oxaliplatin-induced overproduction of reactive oxygen species (ROS) but also reversed parthanatos caused by oxaliplatin. In conclusion, our results indicate that oxaliplatin inhibits OSCC by activating PARP1-mediated parthanatos through increasing the production of ROS.

## INTRODUCTION

Oral squamous cell carcinoma (OSCC), belonging to a malignant group of tumors, is one of the most prevailing subtypes of head and neck squamous cell carcinoma (HNSCC), representing the sixth most common cancer worldwide [1–3]. Unfortunately, as a result of its diverse etiologies and biological heterogeneity, OSCC is still characterized by high morbidity and relatively low overall 5-year survival rates and remains to be a serious global public health issue [1–3]. In addition to surgery and radiotherapy, chemotherapy is extensively regarded as one of the most effective clinical treatments for OSCC patients, among the available treatment methods [4].

**Figure 1:**
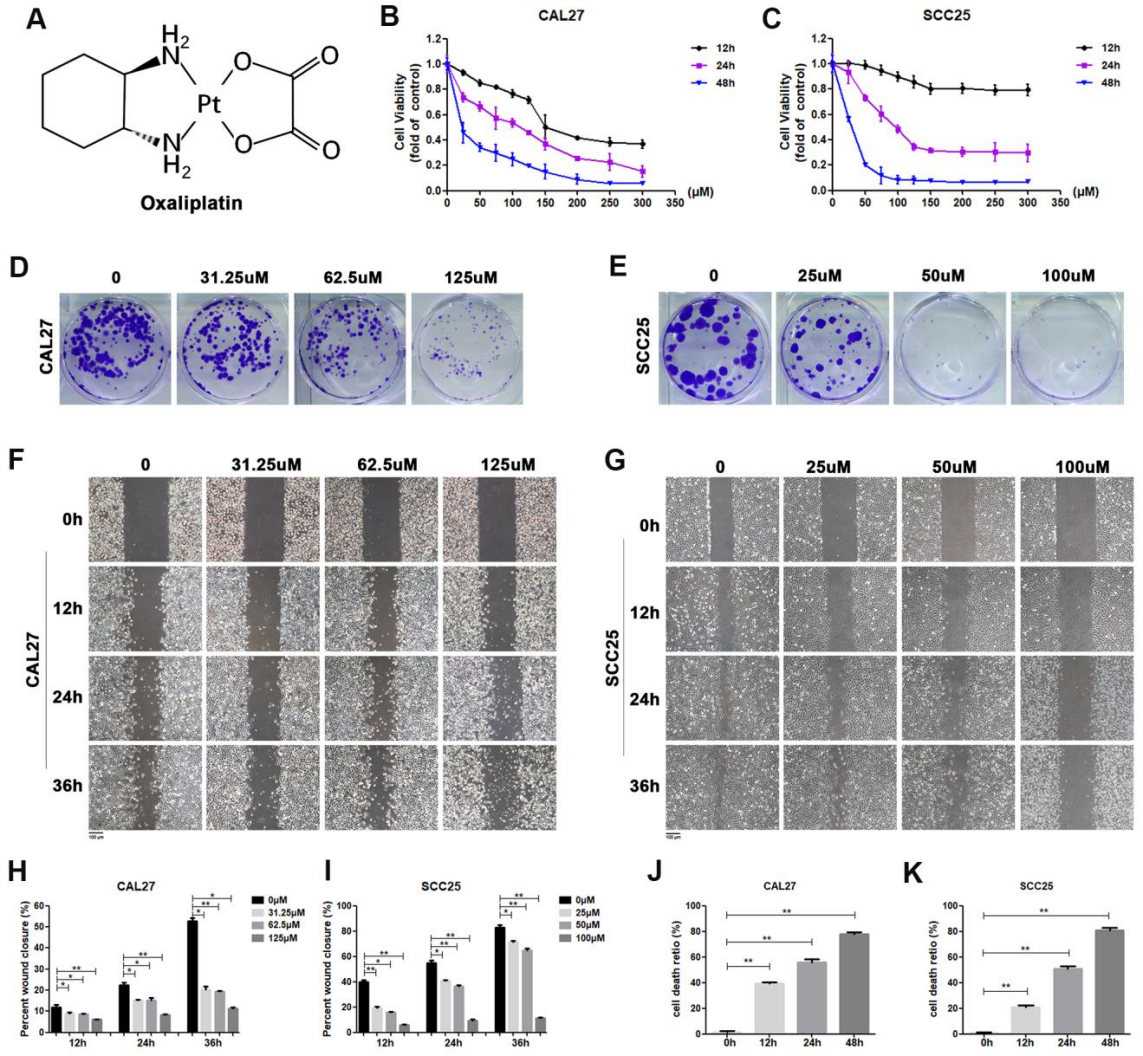
**Oxaliplatin inhibited cell viability, migration and cloning formation of OSCC cells and induced cell death *in vitro*.** (**A**) Chemical structure of oxaliplatin. (**B**, **C**) Cell viability of CAL27 and SCC25 cells treated with different concentrations of oxaliplatin for 12, 24, and 48 hours. (**D**, **E**) The colony formation assay of CAL27 and SCC25 cells treated with oxaliplatin for 10 days. (**F**–**I**) The wound healing detection of CAL27 and SCC25 cells treated with different concentrations of oxaliplatin for 12, 24, and 36 hours. (**J**, **K**) The cell death ratio based on LDH release assay of CAL27 and SCC25 cells treated with 125 μM and 100 μM oxaliplatin. * p<0.05, **p<0.01.

Platinum-based chemotherapy is still the gold standard for treating different solid tumors, although its history can be traced back to 1965, when Rosenborg accidentally discovered an anti-mitotic activity platinum (II) complex that was transmitted in the growth of bacteria in saline in a chamber equipped with a set of platinum electrodes [5, 6]. Oxaliplatin is a relatively new third generation platinum analogue containing diaminocyclohexane (Figure 1A), which was first patented in 1976 and approved for medical use in 1996 [7]. Oxaliplatin is also an alkylating agent and is widely used in the treatment of multiple malignancies, including pancreatic cancer [8], colorectal cancer [9], gastroesophageal cancer [10], ovarian cancer [11], breast cancer [12], non-small cell lung cancer [13], gall bladder cancer [14], non-Hodgkin's lymphoma [15], and HNSCC [16, 17]. However, there are few reports on the effect of oxaliplatin in the treatment of OSCC.

Oxaliplatin-induced cytotoxicity is mainly caused by inserting platinum adducts into nuclear DNA to cause DNA damage [18, 19], promoting mitotic cell cycle arrest and inducing apoptosis [20, 21]. Recent studies have also shown that oxaliplatin can induce an increased formation of reactive oxygen species (ROS) [22, 23], mitochondrial membrane depolarization [24], and over-activation of poly (ADP-ribose) polymerase 1 (PARP1) [25, 26], which is closely related to a new form of cell death, parthanatos. Different from other forms of cell death, such as apoptosis, pyroptosis, and autophagic cell death, parthanatos is a unique, highly orchestrated form of cell death that occurs through the excessive activation of the nuclear DNA nick sensor enzyme, PARP1. Mechanistically, being a distinct cell death pathway, parthanatos is associated with a series of biochemical events, such as hyperactivation of PARP1, synthesis and accumulation of PAR polymer, mitochondrial depolarization, and interaction and nuclear translocation of the mitochondrial protein apoptosis-inducing factor (AIF) and the macrophage migration inhibitory factor (MIF) [27–29]. Interestingly, there is increasing evidence showing that an alkylating agent can induce pathanatos [23, 27–30]. Being an alkylating agent, whether oxaliplatin can induce parthanatos in OSCC is still unknown.

The aim of the present study was to examine whether oxaliplatin can cause parthanatos in OSCC, so as to provide a new theoretical basis for the clinical application of chemotherapy drugs in the treatment of OSCC. Increased production of ROS, significant up-regulation of PARP1, and interaction and nuclear translocation of the AIF and MIF were found in oxaliplatin-treated OSCC cell lines, CAL27 and SCC25. Above all, our results showed that oxaliplatin might induce PARP1 mediated parthanatos in OSCC by increasing the production of ROS.

## RESULTS

### Oxaliplatin inhibited cell viability, migration, and cloning formation of OSCC cells and induced cell death *in vitro*

In order to determine the effect of oxaliplatin on cell viability, CAL27 cells and SCC25 cells were treated with different concentrations of oxaliplatin for 12, 24, and 48 hours. As shown in Figure 1B, 1C, cell viability was inhibited by oxaliplatin in a dose and time dependent manner when compared to the control group. The half maximal inhibitory concentration (IC50) value of oxaliplatin at 24 hours was about 125 μM in CAL27 cells and 100 μM in SCC25 cells, so these concentrations and half and quarter of these concentrations were chosen for use in subsequent studies. Additionally, to evaluate the impact of oxaliplatin on cell cloning, the cloning formation assay was performed. Results showed that the number of cell clones of CAL27 and SCC25 cells was decreased in the presence of oxaliplatin in a dose-dependent manner (Figure 1D, 1E), suggesting that oxaliplatin could inhibit the clonogenic ability of OSCC cells. Meanwhile, oxaliplatin was found to have a suppression effect on migration of CAL27 cells and SCC25 cells in a dose-dependent manner (Figure 1F–1I). To further study the effects of oxaliplatin on cell death in OSCC cells, the cell death ratio was detected through lactate dehydrogenase (LDH) release assay. The results showed that the cell death ratio increased with the passage of time in CAL27 cells treated with 125 μM oxaliplatin and SCC25 cells treated with 100 μM oxaliplatin (Figure 1J, 1K). All these results indicated that oxaliplatin can not only inhibit the biological activity of OSCC cells, but it can also cause cell death.

### Oxaliplatin inhibited tumor growth of OSCC and caused upregulation of PARP1 *in vivo*

**Figure 2:**
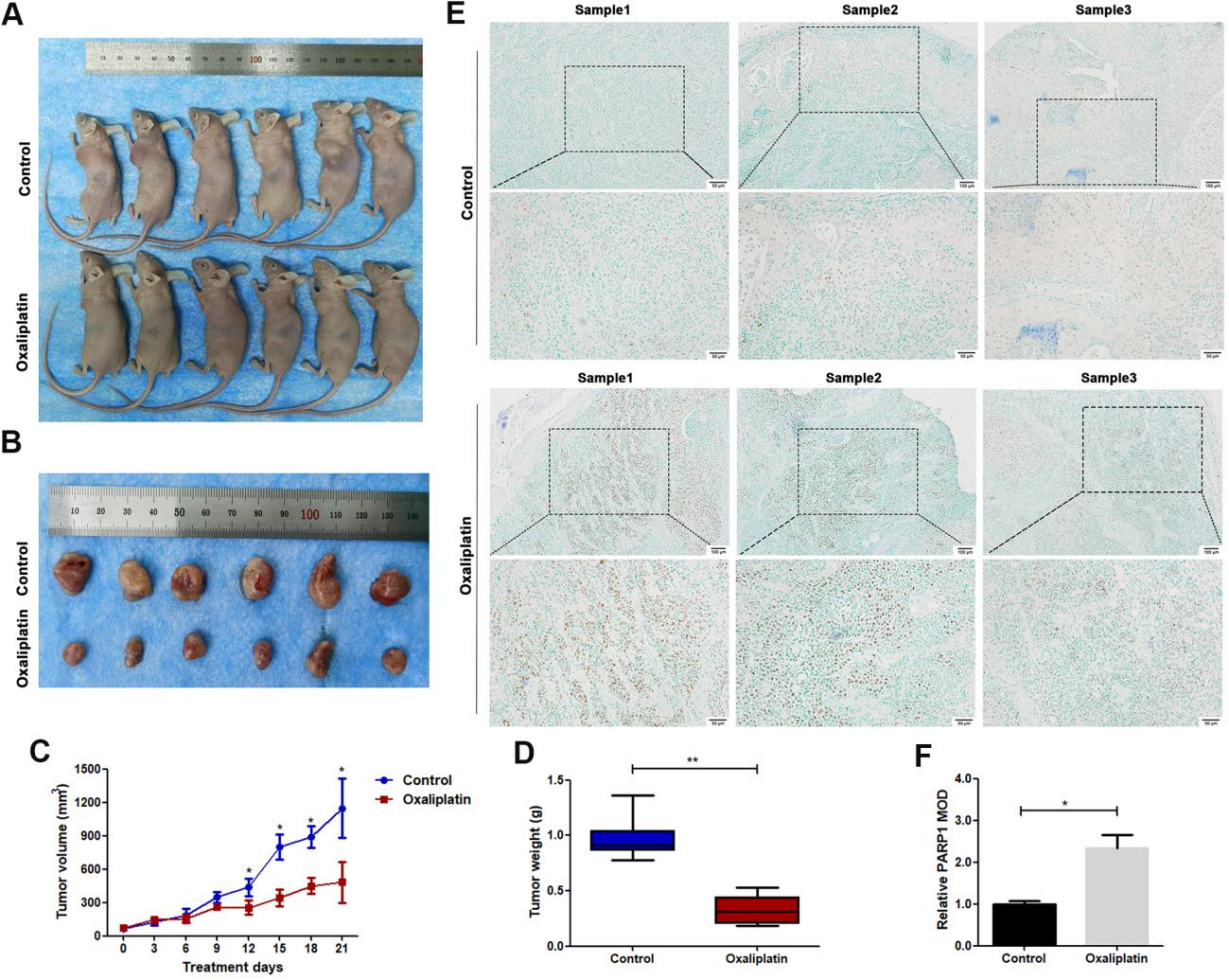
**Oxaliplatin inhibited tumor growth of OSCC and caused upregulation of PARP1 *in vivo*.** (**A**) The xenograft models using BALB/c mice are shown. (**B**) The tumors derived from BALB/c mice are shown. (**C**) The volumes of tumors during the 21 days of treatment. (**D**) The average weight of tumors after treatment with oxaliplatin or PBS for 21 days. (**E**) Representative images of PARP1immunohistochemical staining of xenografts in control and oxaliplatin treatment group. (**F**) Relative mean optical density (MOD) of PARP1 in control and oxaliplatin treatment group. * p<0.05, **p<0.01.

The *in vivo* anticancer effect of oxaliplatin was studied using a nude mouse subcutaneously implanted with CAL27 cells. The average volume of tumors was markedly smaller in the oxaliplatin group than in the control group (Figure 2A–2C). The average weight of tumors was also lesser in the oxaliplatin group than in the control group (Figure 2D). Furthermore, immunohistochemical staining showed that oxaliplatin treatment resulted in upregulation of PARP1 as compared with that in the control group (Figure 2E, 2F). Therefore, these results indicated that oxaliplatin inhibited the growth of xenograft OSCC cells *in vivo*, which was accompanied by the upregulated expression of PARP1.

### Oxaliplatin caused mitochondrial damage in OSCC cells

**Figure 3:**
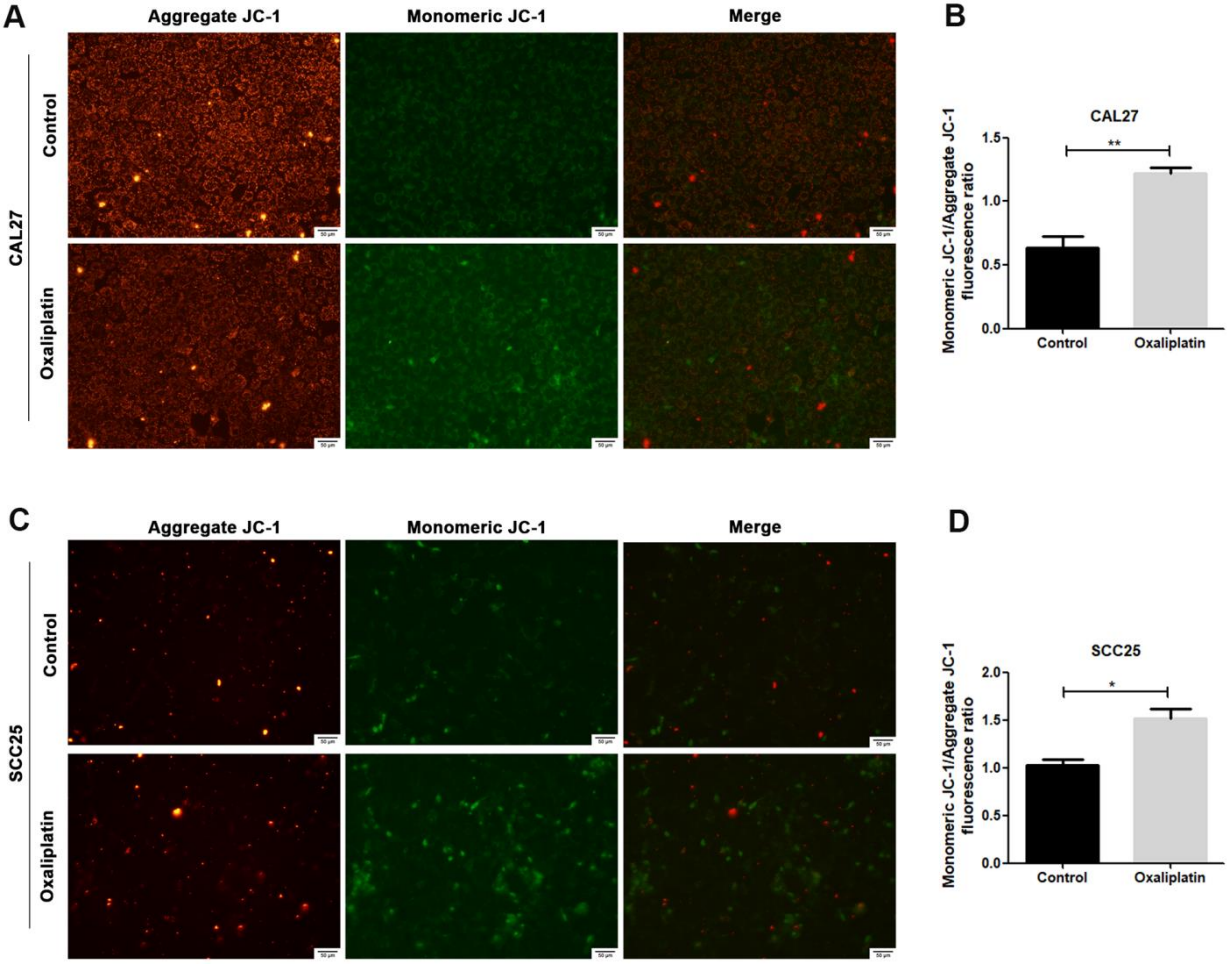
**Oxaliplatin caused mitochondrial damage in OSCC cells.** (**A, C**) Representative images of JC-1 staining in CAL27 and SCC25 cells treated with oxaliplatin. (**B, D**) The fluorescence ratio of monomeric JC-1/ aggregate JC-1 in CAL27 and SCC25 cells treated with oxaliplatin. * p<0.05, **p<0.01.

JC-1 assay was used to detect whether oxaliplatin could cause mitochondrial damage in OSCC cells. Generally, JC-1 accumulates in mitochondria and presents high red fluorescence (aggregate JC-1). But when mitochondria are damaged, JC-1 tends to exist in the cytoplasm and presents green fluorescence due to a decrease in the mitochondria membrane potential (monomeric JC-1). As shown in Figure 3A–3D, when CAL27 and SCC25 cells were treated with oxaliplatin, monomeric JC-1 was increased and aggregate JC-1 was decreased, indicating that oxaliplatin induced depolarization and damage in the mitochondrion among OSCC cells.

### Oxaliplatin led to the upregulation of PARP1 and nuclear translocation of AIF in OSCC cells

**Figure 4:**
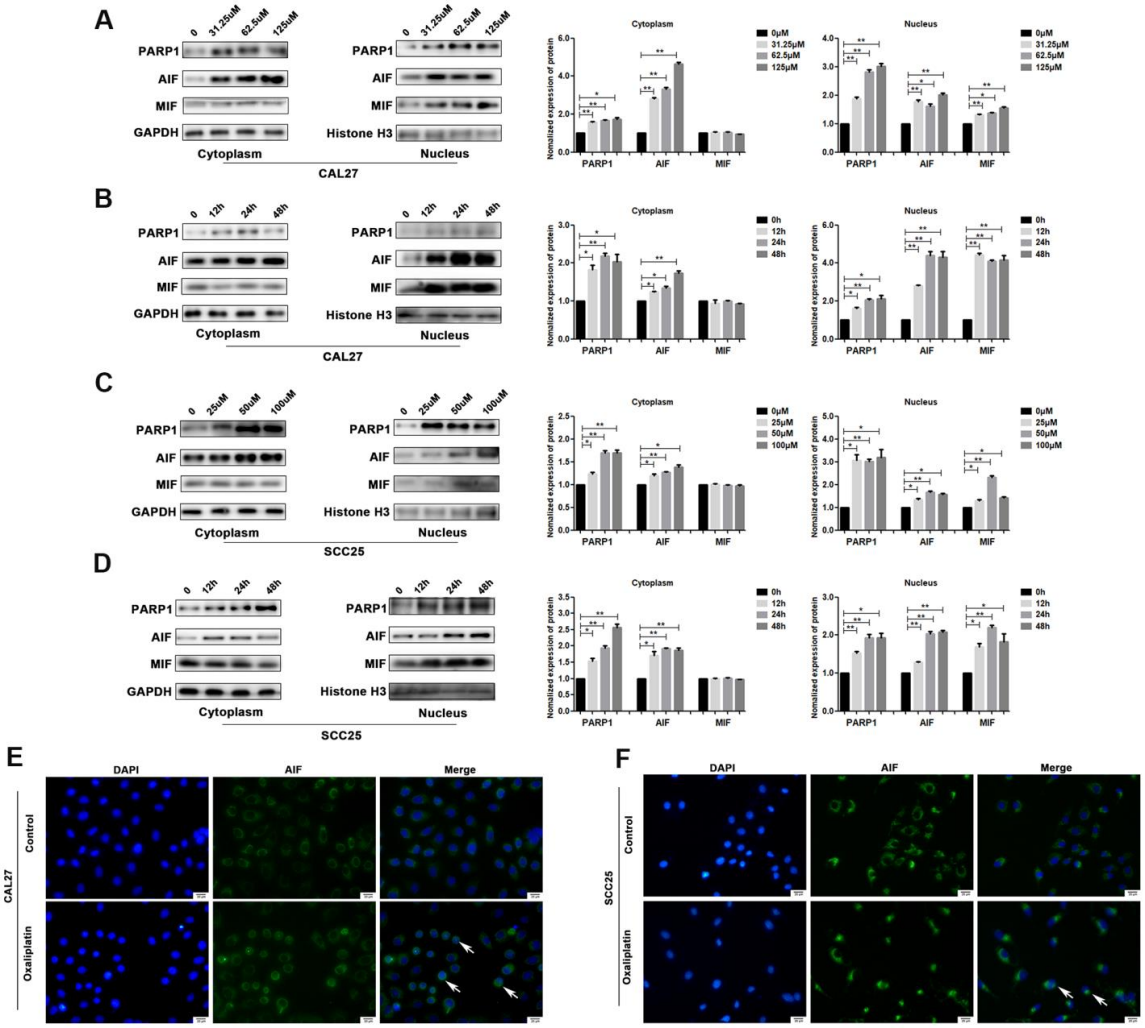
**Oxaliplatin caused the upregulation of nuclear PARP1, AIF and MIF, and nuclear translocation of AIF in OSCC cells.** (**A**) The protein levels of PARP1, AIF, and MIF in the nucleus and cytoplasm of CAL27 cells treated with 0, 31.25, 62.5, and 125 μM oxaliplatin for 48 hours. (**B**) The protein level of PARP1, AIF, and MIF in the nucleus and cytoplasm of CAL27 cells treated with 125 μM oxaliplatin for 0, 12, 24, and 48 hours. (**C**) The protein levels of PARP1, AIF and MIF in the nucleus and cytoplasm of SCC25 cells treated with 0, 25, 50, and 100 μM oxaliplatin for 48 hours. (**D**) The protein levels of PARP1, AIF, and MIF in the nucleus and cytoplasm of SCC25 cells treated with 100 μM oxaliplatin for 0, 12, 24, and 48 hours. (**E**, **F**) Representative images of AIF immunofluorescence staining in CAL27 and SCC25 cells treated with oxaliplatin. Arrows indicates typical cells with AIF nuclear translocation. * p<0.05, **p<0.01.

PARP1, AIF and MIF are important effectors in parthanatos; thus, we detected their expression levels in oxaliplatin-treated OSCC cells. The results of western blot showed that the protein levels of PARP1, AIF and MIF were much higher in the nucleus of oxaliplatin- treated CAL27 cells as compared to those in the control group (Figure 4A, 4B). In addition, the degree of PARP1, AIF, and MIF up-regulation was increased in the nucleus with an increase in the oxaliplatin concentration and incubation time (Figure 4A, 4B). Similar changes were also observed in oxaliplatin-treated SCC25 cells (Figure 4C, 4D). To further detect the nuclear translocation of AIF, we used cell immunofluorescence to detect the distribution of AIF protein in OSCC cells. As shown in Figure 4E, 4F, more amount of AIF was located in the nucleus of CAL27 cells and SCC25 cells when they were treated with oxaliplatin, which proved that oxaliplatin promoted nuclear translocation of AIF.

### Down-regulating the expression of PARP1 reversed oxaliplatin-induced parthanatos in OSCC cells

**Figure 5:**
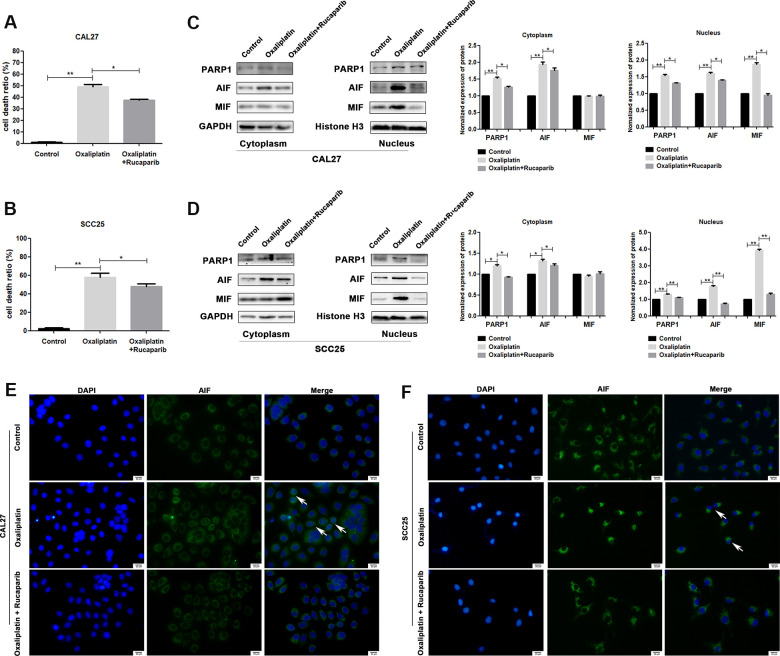
**PARP1 inhibitor rucaparib reversed oxaliplatin-induced parthanatos in OSCC cells.** CAL27 and SCC25 cells were treated with or without oxaliplatin or oxaliplatin plus rucaparib. (**A**, **B**) The cell death ratio based on LDH release assay of CAL27 and SCC25 cells. (**C**, **D**) The protein levels of PARP1, AIF and MIF in the nucleus and cytoplasm of CAL27 cells. (**E**, **F**) Representative images of AIF immunofluorescence staining in CAL27 and SCC25 cells. Arrows indicates typical cells with AIF nuclear translocation. * p<0.05, **p<0.01.

**Figure 6:**
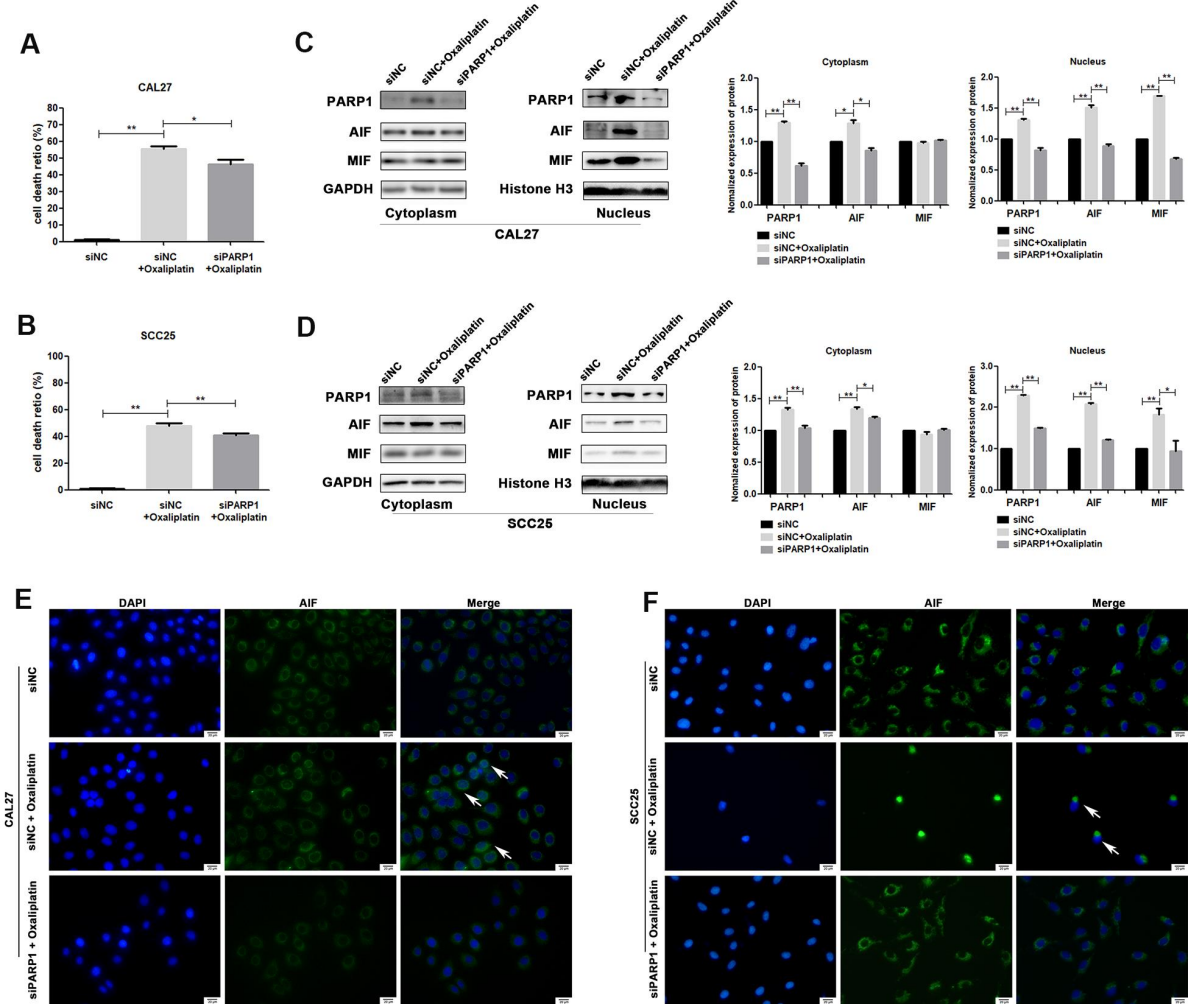
**siRNA against PARP1 reversed oxaliplatin-induced parthanatos in OSCC cells.** CAL27 and SCC25 cells were transfected with siRNA against PARP1 (siPARP1) or non-targeting siRNA (siNC), and they were further treated with or without oxaliplatin. (**A**, **B**) The cell death ratio based on LDH release assay of CAL27 and SCC25 cells. (**C**, **D**) The protein level of PARP1, AIF and MIF in the nucleus and cytoplasm of CAL27 cells. (**E**, **F**) Representative images of AIF immunofluorescence staining in CAL27 and SCC25 cells. Arrows indicates typical cells with AIF nuclear translocation. * p<0.05, **p<0.01.

To further confirm whether oxaliplatin inhibits the development of OSCC by inducing PARP1-mediated parthanatos, we treated CAL27 and SCC25 cells with rucaparib (an inhibitor of PARP1) to interfere with the activation of PARP1 induced by oxaliplatin. By measuring the release of LDH, we found that rucaparib reversed the increase in cell mortality induced by oxaliplatin treatment in OSCC cells (Figure 5A, 5B). Then the expression levels of PARP1, AIF, and MIF were detected by western blot or immunofluorescence. The results of western blot showed that rucaparib treatment inhibited the upregulation of PARP1, AIF and MIF induced by oxaliplatin in the nucleus (Figure 5C, 5D), as well as the nuclear translocation of AIF (Figure 5E, 5F). In addition, we transfected CAL27 and SCC25 cells with siRNA against PARP1 to downregulate the expression of PARP1 induced by oxaliplatin, and we obtained the similar trends: knockdown of PARP1 reversed the increased cell death rate (Figure 6A, 6B), the expression of AIF and MIF in nucleus (Figure 6C, 6D), and the nuclear translocation of AIF induced by oxaliplatin treatment (Figure 6E, 6F). Thus, our results suggested that oxaliplatin induced the death of OSCC cells by activating PARP1-mediated parthanatos.

### Oxaliplatin caused ROS overproduction and oxidative stress in OSCC cells

**Figure 7:**
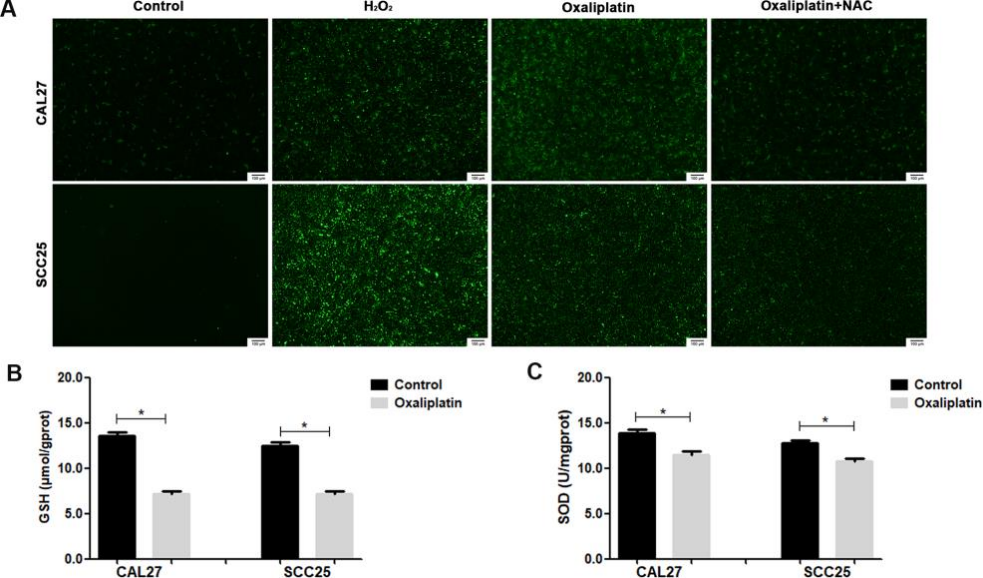
**Oxaliplatin caused ROS overproduction and oxidative stress in OSCC cells.** (**A**) Representative images of ROS fluorescence in CAL27 and SCC25 cells treated with H_2_O_2_, oxaliplatin, or oxaliplatin plus antioxidant NAC. (**B**) The SOD activity in CAL27 and SCC25 cells treated with or without oxaliplatin. (**C**) The GSH concentration in CAL27 and SCC25 cells treated with or without oxaliplatin. * p<0.05.

By detecting ROS production in CAL27 and SCC25 cells via immunofluorescence, we found that oxaliplatin treatment significantly increased the production of ROS in OSCC cells (Figure 7A). At the same time, the superoxide dismutase (SOD) activity and glutathione (GSH) concentration were detected in oxaliplatin-treated CAL27 and SCC25 cells, which reflected the antioxidant capacity of cells. The results showed that oxaliplatin caused a significant decrease in SOD activity and GSH concentration (Figure 7B, 7C), suggesting that oxaliplatin impaired the antioxidant capacity of OSCC cells. All these results proved that oxaliplatin caused ROS overproduction and oxidative stress in OSCC cells.

### Oxaliplatin-induced parthanatos occurred through the overproduction of ROS

**Figure 8:**
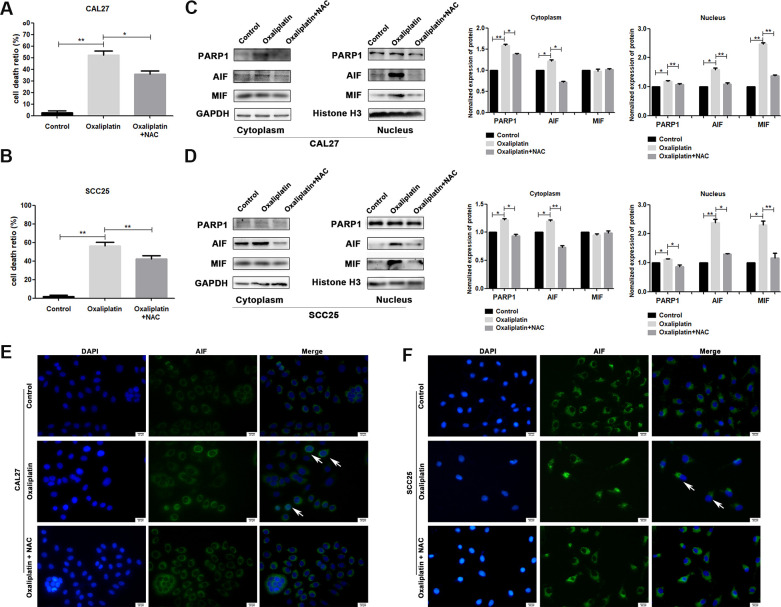
**NAC reversed oxaliplatin-induced parthanatos in OSCC cells.** CAL27 and SCC25 cells were pretreated with or without antioxidant NAC for 1 hour, and then they were treated with oxaliplatin. (**A**, **B**) The cell death ratio based on LDH release assay of CAL27 and SCC25 cells. (**C**, **D**) The protein levels of PARP1, AIF and MIF in the nucleus and cytoplasm of CAL27 cells. (**E**, **F**) Representative images of AIF immunofluorescence staining in CAL27 and SCC25 cells. Arrows indicates typical cells with AIF nuclear translocation. * p<0.05, **p<0.01.

To investigate whether ROS affected oxaliplatin-induced parthanatos, we pretreated CAL27 and SCC25 cells with antioxidant NAC for 1 hour. As shown in Figure 7A, NAC effectively inhibited the production of ROS, which was induced by oxaliplatin. Then, by measuring LDH release, we found that inhibiting the production of ROS can reduce the cell death induced by oxaliplatin in CAL27 and SCC25 cells (Figure 8A, 8B). Similarly, western blot results showed that NAC significantly inhibited oxaliplatin-induced overexpression of PARP1, AIF, and MIF in the nucleus of OSCC cells (Figure 8C, 8D). The results of immunofluorescence showed that NAC repressed nuclear translocation of AIF mediated by oxaliplatin (Figure 8E, 8F). Thus it could be seen that the production of ROS played an important role in oxaliplatin-induced parthanatos.

## DISCUSSION

Previous studies have shown that oxaliplatin has effective antitumor effects *in vivo* and *in vitro*, and its safety is better than that of cisplatin [31]. Moreover, oxaliplatin may be an innovative and promising drug because of its ability to treat the tumors that are resistant to cisplatin and carboplatin [32]. This study confirmed that oxaliplatin not only significantly inhibited the proliferation, clone formation, and migration of OSCC cells *in vitro*, but it also inhibited the tumorigenesis of OSCC cells in nude mice. These results indicate that oxaliplatin can inhibit the occurrence and development of OSCC, which provides a theoretical basis for the clinical application of oxaliplatin.

Oxaliplatin has been reported to inhibit cancer through a variety of mechanisms. For example, several studies prove that oxaliplatin can induce DNA damage in cancer cells, just like other platinum-based chemotherapy drugs. In addition, oxaliplatin can interfere with the cell cycle, promote apoptosis, and induce autophagy in tumor cells [33–35]. Our study confirmed that oxaliplatin significantly increased the expression of PARP1, further promoted the nuclear translocation of AIF, and eventually led to the parthanatos type of death in OSCC cells. In addition, this effect of oxaliplatin was enhanced with an increase in drug concentration and incubation time. These results revealed a new mechanism for oxaliplatin to inhibit OSCC, which deepens our understanding of the anti-tumor effects of oxaliplatin. In fact, parthanatos has been proved to exist in many physiological and pathological processes, such as diabetes [36], cerebral ischemia [37], and neurodegeneration [27, 38]. Additionally, several studies have reported that a few other drugs can also play an anti-cancer effect through parthanatos; for example, deoxypodophyllotoxin inhibits glioma through parthanatos [39]. Therefore, it is very important to study the mechanism of parthanatos for antitumor research.

It is worth noting that many studies on parthanatos have found that PARP1 plays a dual role in the process of cell death [40]. PARP1 is an important cellular ribozyme that is involved in several physiological processes, such as DNA repair [41]. Previous studies proved that the suppression of PARP1 can inhibit the repair process of DNA damage, thus enhancing the sensitivity of the tumor to chemoradiotherapy [42–44]. Thus, PARP1 inhibitors are promising and have been applied to cancer treatment. For instance, olaparib, rucaparib, niraparib, and talazoparib have been approved for clinical applications in ovarian cancer, breast cancer, and pancreatic cancer [45–47]. However, in this study, we found that oxaliplatin can induce overactivation of PARP1, leading to release of AIF, and then AIF interacted with MIF and was transferred into the nucleus, resulting in parthanatos in OSCC cells, which is similar to a previous study on gliomas [48]. Also, on inhibiting the activation of PARP1 by using PARP1 inhibitor or siRNA, the lethal effect of oxaliplatin on OSCC cells was significantly reversed. Therefore, overactivation of PARP1 is an important part in the process of oxaliplatin-induced parthanatos, and it may also be a potential effective anti-tumor method.

ROS are a type of single electron reduction products of oxygen produced in cells, whose excessive accumulation can cause damage to the cells [49]. Our results showed that oxaliplatin induced the production of ROS and impaired the antioxidant capacity of OSCC cells, which presented as the decrease in SOD activity and GSH concentration. In addition, antioxidant NAC not only relieved oxaliplatin-induced overproduction of ROS, but it also inhibited the overexpression of PARP1 and nuclear translocation of AIF caused by oxaliplatin, suggesting that NAC reversed oxaliplatin-induced parthanatos. These results indicated that the production of ROS plays an important role in the process of parthanatos induced by oxaliplatin, which was consistent with some previous studies [23, 50, 51].

**Figure 9:**
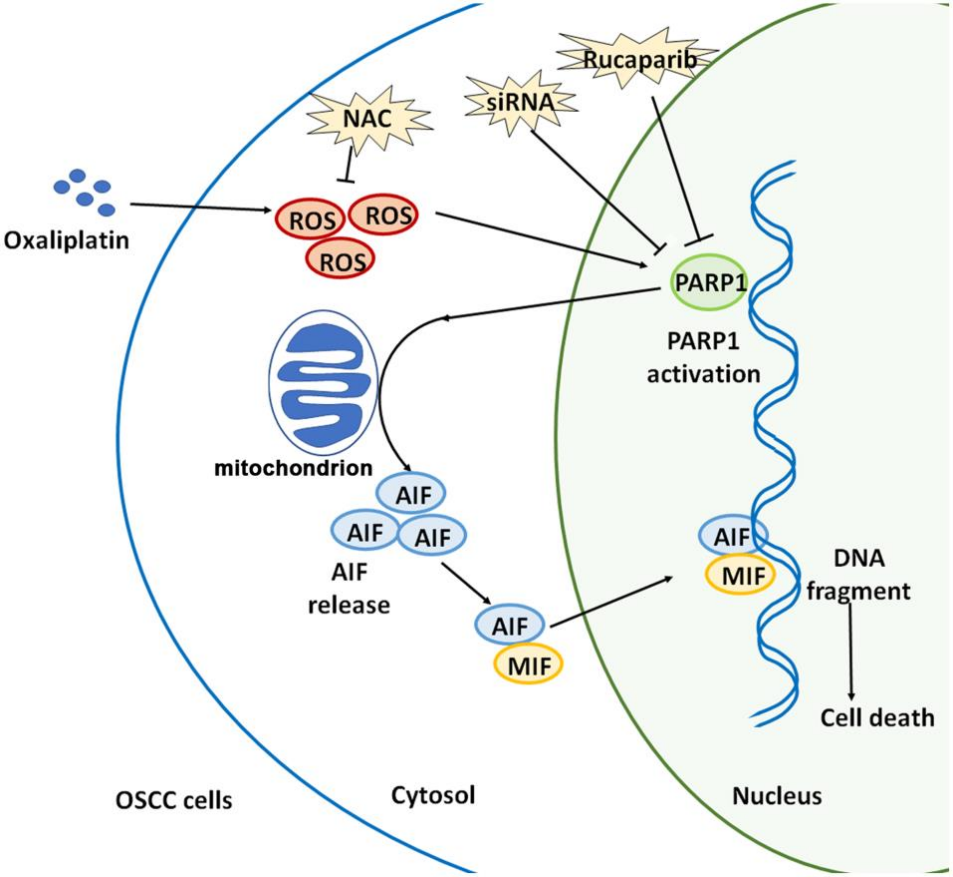
**Schematic illustration of how oxaliplatin induces the PARP1-mediated parthanatos in OSCC cells by increasing the production of ROS.** Oxaliplatin induces the overproduction of ROS and then activates PARP1, which further induces mitochondrial depolarization and AIF release and promotes the nuclear translocation of AIF and MIF, finally inducing cell death (parthanatos).

Collectively, oxaliplatin is a novel third-generation platinum-based chemotherapeutic drug, and oxaliplatin can completely exert its clinical therapeutic effects only when its anti-tumor mechanism is elucidated. This study confirmed that oxaliplatin can increase the production of ROS, and then it can induce the overactivation of PARP1, the depolarization of mitochondria, and the nuclear translocation of AIF and MIF, leading to parthanatos in OSCC cells. (Figure 9). This article is expected to provide a theoretical basis for optimizing chemotherapy for OSCC, and indicate a direction for future studies, which includes continued focus on how to enhance the oxaliplatin-induced parthanatos effect to treat tumors.

## MATERIALS AND METHODS

### Cell culture and reagents

Both human CAL27 and SCC25 cell lines were obtained from Shanghai Cell Bank of Chinese Academy of Sciences (Shanghai, China). The cells were cultured in DMEM/F12 medium containing 10% fetal bovine serum and 1% penicillin-streptomycin at 37° C in a 5% CO2 humidified incubator. The medium was changed every 2 days. Cells in the mid-log phase were used in the experiment. Oxaliplatin (ab141054) was purchased from Abcam (MA, USA). N-Acetyl Cysteine (NAC, 3 mM) and rucaparib (1mM) were purchased from MedChemExpress (MCE, NJ, USA).

### Cell viability assay

CAL27 and SCC25 cells were seeded into 96-well plates and cultured for 24 hours, and then they were treated with different concentrations of oxaliplatin (0, 25, 50, 75, 100, 125, 150, 200, 250, and 300 μM). After 12, 24, and 48 hours, the cell viability was detected with the cell counting Kit-8 kit (CCK8) according to the manufacturer’s instruction. Briefly, each well was added with 10 μL of CCK8 working solution for 1 hour, and the absorbance of each well was measured with a microplate reader at 450 nm wavelength.

### Colony formation assay

CAL27 and SCC25 cells were seeded into a 6-well plate and cultured for 24 hours, and then they were treated with different concentrations of oxaliplatin for about 10 days. Then, the medium was removed and the cells were washed with PBS, fixed with 4% paraformaldehyde, and stained with crystal violet.

### Wound healing assay

CAL27 and SCC25 cells were seeded into 6-well plates. When the cell density reached 100%, a sterile 200 μl pipette tip was used to make a "wound" on the culture surface and then the cells were washed by PBS to remove the sloughed cells. Cell culture was carried out in a medium containing 1% fetal bovine serum and different concentrations of oxaliplatin for the next 36 hours. At four time points (0, 12, 24, and 36 hours), images of the orifice plate were obtained using a microscope camera system.

### Lactate dehydrogenase (LDH) release cell death assay

CAL27 and SCC25 cells were seeded into 96-well plates and cultured for 24 hours, and then they were treated with 125/100 μM oxaliplatin respectively. At four time points (0, 12, 24, and 48 hours), the LDH cytotoxicity detection kit (Beyotime, Shanghai, China) was used to detect cell mortality. The absorbance at 490 nm was measured, and cell mortality was calculated with the following formula: mortality (%) = (absorbance of processed sample - absorbance of sample control hole) / (absorbance of cell maximum enzyme activity - absorbance of sample control hole) × 100.

### Mitochondrial membrane potential (JC-1) assay

CAL27 and SCC25 cells treated with and without oxaliplatin were stained with JC-1 (Beyotime, Nanjing, China) according to the manufacturer’s instructions, and then the cells were observed under a fluorescence microscope (Olympus, Tokyo, Japan).

### Measurement of intracellular reactive oxygen species (ROS)

Intracellular ROS generation was measured using the ROS Detection Kit (KeyGEN BioTECH, Nanjing, China) according to the manufacturer’s instructions. Briefly, after being treated with or without oxaliplatin, cells were washed in DMEM/F12 medium for three times, and then they were incubated with 10 μM DCF-DA at 37° C for 20 minutes. After being washed with PBS, cells were observed under a fluorescence microscope (Olympus, Tokyo, Japan).

### Measurement of intracellular glutathione (GSH) and superoxide dismutase (SOD)

CAL27 and SCC25 cells were treated with PBS or oxaliplatin, and then they were collected and homogenized in ice-cold PBS. After being centrifuged for 15 minutes at 12,000 rpm at 4° C, the supernatants were collected and used for the analysis. Total GSH and SOD detection assays were performed using the Micro Reduced Glutathione (GSH) Assay Kit (Nanjing Jiancheng, Nanjing, China) and Total SOD Detection Kit (Nanjing Jiancheng, Nanjing, China) according to the manufacturer's protocol.

### Immunofluorescence staining

CAL27 and SCC25 cells were fixed with 4% formaldehyde, permeabilized with 0.5% Triton X-100, blocked with 1% BSA, and incubated with the primary antibody (AIF, 1:500 dilution, Abcam, MA, USA) overnight at 4° C, followed by incubation with the secondary antibody (FITC-conjugated goat anti-rabbit IgG, 1:200 dilution, Abcam, MA, USA) for 1 hour at room temperature, and then they were incubated with DAPI for 5 minutes. Finally, the cells were observed under fluorescent microscope (Olympus, Tokyo, Japan).

### Transfection of small interfering RNA (siRNA)

CAL27 and SCC25 cells were inoculated into a culture dish and cultured for 24 hours before transfection with siRNA. Transfection of siRNA was performed by using Lipofectamine 2000 (Invitrogen, USA) according to the vendor’s protocol. The sequence of siRNA against PARP1 (siPARP1) was 5′-AAGCCAUGGUGGAGUAUGATT-3′. The non-targeting siRNA (siNC) was used as the control.

### Establishment of a mouse xenograft model

All animal studies were approved by the Institutional Animal Care and Use Committee (IACUC) of Shandong University. Athymic nude BALB/c female mice (aged 4-6 weeks, weight 16±4g) purchased from Jinan Pengyue Laboratory Animal Breeding Co. Ltd., were housed in a specific pathogen-free environment under the condition of 12-hour light/12-hour dark cycle and free food and water, and they were acclimatized to their surroundings for 3 days. Mice were randomly divided into two groups (n=6) and were injected subcutaneously in the left flank with 3×10^6^ CAL27 cells in 100 μl PBS. The therapeutic experiments were started when the tumor size reached about 100 mm^3^ after 5 to 7 days. Subjects were injected intraperitoneally (once every two days) with oxaliplatin (5 mg/kg in PBS, oxaliplatin group) or same volume of PBS (control group). After 21 days, mice were euthanized, and their xenografts were harvested and weighed. The tumor size was measured using a slide caliper, and the tumor volume was calculated using the following formula: 0.5×A×B^2^, in which A is the length of the tumor and B is the width. Tumor tissues were fixed immediately with 4% paraformaldehyde for subsequent analysis.

### Immunohistochemical (IHC) staining

The expression levels of PARP1 in different xenografts were detected at the protein level by IHC staining. Briefly, xenografts embedded in paraffin were cut into 5 μm sections and fixed onto slides. Then, the slides were deparaffinized, hydrated, and microwave-treated for antigen retrieval. The slides were incubated with the PARP1antibody (1:200 dilution, Abcam, MA, USA) overnight, and then were incubated with secondary antibody, horseradish peroxidase (HRP)-conjugated goat anti-rabbit IgG (1:200 dilution, Abcam, MA, USA) at 37° C in dark for 1 hour. Satisfactory immunostaining was acquired in the presence of diaminobenzidine (DAB) (Sigma, MO, USA). Finally, the slides were counterstained with methyl green, subjected to gradient alcohol and xylene dehydration, sealed with neutral gum, and observed under a fluorescent microscope (Olympus, Tokyo, Japan).

### Western blot analysis

To obtain the nuclear and cytoplasmic protein of cells, the Nuclear and Cytoplasmic Protein Extraction Kit (Bosterbio, Wuhan, China) was used according to the manufacturer’s instructions. Protein concentrations were determined using BCA protein assays (Beyotime, Beijing, China). The protein was separated by 12 % SDS-PAGE gel electrophoresis and transferred to a polyvinylidene fluoride (PVDF) membrane by electrolysis. After being blocked with 5% BSA in TBST for 1 hour at room temperature, the membranes were incubated with the primary antibodies overnight at 4° C. Here the following specific primary antibodies were used as follows: PARP1 (1:2000 dilution; Abcam, MA, USA), AIF (1:1000 dilution, Abcam, MA, USA), MIF (1:1000 dilution, Abcam, MA, USA), Histone-3 (1:500 dilution, Abcam, MA, USA), and GAPDH (1:1000 dilution, Abcam, MA, USA). Then the membranes were washed three times with TBST, and they were incubated with the corresponding secondary antibodies conjugated with horseradish peroxidase for 1 hour. The membranes were washed three times with TBST for 15 minutes each time. Finally, the immune response zones were detected using an ECL detection system (SmartChemi 420, Beijing, China). The results were analyzed by Image J software.

### Statistical analysis

All primary data were presented as the mean ± SEM. P values of less than 0.05 were considered statistically significant. Unless otherwise indicated, statistical evaluation was carried out by Student’s t test between two groups and by one-way analysis of variance (ANOVA) followed by post hoc comparisons with the Bonferroni test using GraphPad Prism software among multiple groups. The experiments for quantification were performed in a blinded fashion. In order to ensure adequate capacity to detect the effect, at least 3 independent tests were performed for all molecular biochemistry studies.
